# Inhibition of Endothelial Inflammatory Response by HT-C6, a Hydroxytyrosol Alkyl Ether Derivative

**DOI:** 10.3390/antiox12081513

**Published:** 2023-07-28

**Authors:** Ana Dácil Marrero, Laura Castilla, Manuel Bernal, Inmaculada Manrique, Joel D. Posligua-García, Federico Moya-Utrera, Cristina Porras-Alcalá, José Luis Espartero, Francisco Sarabia, Ana R. Quesada, Miguel Ángel Medina, Beatriz Martínez-Poveda

**Affiliations:** 1 Departamento de Biología Molecular y Bioquímica, Facultad de Ciencias, Universidad de Málaga, Andalucía Tech, 29071 Málaga, Spain; anadacil@uma.es (A.D.M.); lauravcr@uma.es (L.C.); mbernal@uma.es (M.B.); imanrique@uma.es (I.M.); joel.posgar@uma.es (J.D.P.-G.); quesada@uma.es (A.R.Q.); medina@uma.es (M.Á.M.); 2 Instituto de Investigación Biomédica de Málaga y Plataforma en Nanomedicina, IBIMA Plataforma BIONAND, 29590 Málaga, Spain; 3 CIBER de Enfermedades Raras (CIBERER), Instituto de Salud Carlos III, 28029 Madrid, Spain; 4 Departamento de Química Orgánica, Facultad de Ciencias, Universidad de Málaga, 29071 Málaga, Spain; fedemu@uma.es (F.M.-U.); cristinaalcala@uma.es (C.P.-A.); frsarabia@uma.es (F.S.); 5 Departamento de Química Orgánica y Farmacéutica, Facultad de Farmacia, Universidad de Sevilla, 41012 Sevilla, Spain; jles@us.es; 6 CIBER de Enfermedades Cardiovasculares (CIBERCV), Instituto de Salud Carlos III, 28029 Madrid, Spain

**Keywords:** hydroxytyrosol, hydroxytyrosol derivatives, inflammatory response, endothelial cells, NF-κB, HT-C6, antioxidant, atherosclerosis

## Abstract

Hydroxytyrosol (HT) is a bioactive phenolic compound naturally present in olives and extra virgin olive oil (EVOO) which is described as an antioxidant, antitumoral and antiangiogenic molecule. Previous studies of semi-synthetic HT-derivatives presented the hydroxytyrosyl alkyl ether HT-C6 as one of the most potent derivatives studied in the context of antioxidant, anti-platelet and antiangiogenic assays, but its direct effect on inflammation was not reported. In this work, we use RT-qPCR measure of gene expression, protein analysis by Western-blot and immunofluorescence techniques, adhesion and migration functional assays and single-cell monitoring of reactive oxygen species (ROS) in order to explore in vitro the ability of HT-C6 to interfere in the inflammatory response of endothelial cells (ECs). Our results showed that HT-C6 strongly reduces the TNF-α-induced expression of vascular cell adhesion molecule 1 (*VCAM1*), intercellular cell adhesion molecule 1 (*ICAM1*), E-selectin (*SELE*), C-C motif chemokine ligand 2 and 5 (*CCL2* and *CCL5*) in HUVECs, impairing the chemotactic and adhesion potential of these cells towards THP-1 monocytes in vitro. In this work, we define a mechanism of action underlying the anti-inflammatory effect of HT-C6, which involves the abrogation of nuclear factor kappa B (NF-κB) pathway activation in ECs. These results, together with the ability of HT-C6 to reduce ROS formation in ECs, point to this compound as a promising HT-derivative to be tested in the treatment of atherosclerosis.

## 1. Introduction

Benefits derived from phenolic compounds present in extra virgin olive oil (EVOO) have been extensively described in the literature, mainly in the context of the Mediterranean Diet and its health-promoting/chemopreventive potential [1,2]. Within this large group of compounds, hydroxytyrosol (HT) is one of the most studied, exhibiting bioactive potential as a potent antioxidant, antitumour and antiangiogenic, among other properties [3,4,5]. In addition to the relevant bioactive properties of these natural compounds, the synthesis of derivatives based on phenolic structures represents a powerful strategy in the search for new drugs with different therapeutic applications, since modification of the natural structure could result in an improvement of the bioactivity and/or in the pharmacokinetics of the parental compound [6]. In the case of HT, the molecular characteristics define an intrinsic polarity, which restricts its use in lipophilic contexts. Different works have described the synthesis and functional validation of more lipophilic HT derivatives, such as HT esters and HT alkyl ethers [7,8]. In fact, our previous studies identified alkylation as an empowering chemical modification of the molecule in the context of angiogenesis inhibition, since the addition of an alkyl residue to HT improved its antiangiogenic activity compared with a panel of HT derivatives containing other chemical moieties [9]. Furthermore, and continuing with these studies, we evaluated a series of HT alkyl ether derivatives with increasing length of the alkyl chain, identifying the semi-synthetic HT-C6 (hydroxytyrosyl hexyl ether) as the most potent compound of the studied series in a panel of angiogenesis inhibition assays in vitro (directly in endothelial cells [ECs]) and in vivo (in physiologic vessel growth) [10].

In addition to the potent antiangiogenic effect of HT-C6, its potential therapeutic activity in atherosclerosis has been proposed based on the anti-platelet properties suggested by in vitro and in vivo studies [11,12]. The inhibitory activity exhibited by HT-C6 in vitro in platelet aggregation assays and in lipopolysaccharide (LPS)-induced prostaglandin E2 (PGE2) and interleukin 1β (IL1β) production, and the global anti-platelet response measured in rats after oral administration of the compound, suggest a role in the prevention of thrombus formation resulted from endothelial dysfunction in early phases of atheroma development [11,12]. HT-C6 emerges from these data as an interesting molecule in vascular damage prevention, although its direct effect on the endothelial compartment has not been explored before.

The endothelium is an essential player in the onset of atherosclerosis, being implicated in the initial phases of the atheroma formation. Indeed, the activation of ECs in arteries upon proinflammatory signals, such as oxidized lipoproteins, tumor necrosis factor-alpha (TNF-α) or LPS, initiates a specific expression program which results in the exposition of adhesion molecules and the secretion of inflammation-related molecules in the endothelium, favoring the recruitment and diapedesis of immune cells to the subendothelial intima layer [13]. The main intracellular signaling axis underlying these phenotypic changes in ECs is the nuclear factor kappa B (NF-κB) pathway, a master regulator of inflammation in several cell types which modulates the expression of thousands of genes, mainly, but not only, implicated in inflammation [14,15]. The activation of NF-κB is promoted by different ligand/receptor switches, such as TNF-α/TNF receptor (TNFR), LPS/Toll-like receptor 4 (TLR4) or interleukin 1 (IL1)/IL1 receptor (IL1R), and two intracellular cascades have been described downstream them: the canonical and the non-canonical, being the canonical pathway the most strongly activated in cultured ECs in response to TNF-α [16,17]. Apart from inflammatory genes, the NF-κB pathway activates the expression of genes involved in multiple cellular functions, such as cell proliferation and apoptosis, embryogenesis and cell response to oxidative stress.

Concomitant to the vascular-preventive role of HT-C6, its antioxidant activity has been previously explored. In an exhaustive study by Pereira-Cano et al., the antioxidant properties of this and other structurally related compounds were evaluated in vitro in polar hydrophilic and non-polar hydrophobic acellular contexts. This study pointed to HT-C6 as the HT-alkyl derivative with the longest alkyl chain in which antioxidant properties were enhanced compared to HT, since derivatives with longer chains did not exhibit increased antioxidant activity [18]. ECs in early atherosclerosis and atheroma onset are challenged by oxidative damage derived from high reactive oxygen species (ROS) levels produced by immune cells, resulting in endothelial dysfunction and promoting the progression of the disease [19,20]. However, despite the evidence of the antioxidant effect of HT-C6, its direct antioxidant activity in a cellular system has not been previously measured, and even less in ECs.

Based on previous evidence about the potential of HT-C6 in the prevention of vascular damage, in this work we explored the putative role of this semi-synthetic HT derivative in the inflammatory response of ECs, evaluating its direct effect in inflammatory molecules expression, its interference with the master NF-κB signaling pathway, and the functional impairment derived from the HT-C6-driven reduced endothelial cell response. Our results clearly show the anti-inflammatory activity of HT-C6 in ECs, evidencing for the first time a molecular mechanism underlying this effect.

## 2. Materials and Methods

### 2.1. HT-C6 Chemical Synthesis

**Figure 1:**
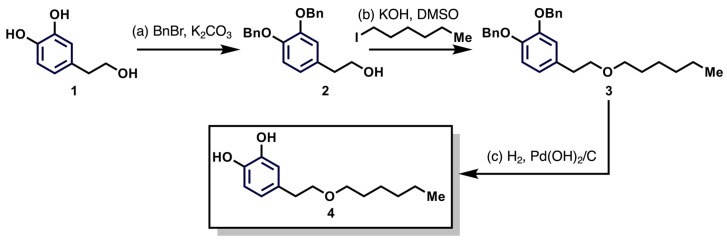
Schematic description of the procedure implemented for HT-C6 synthesis. Compound **1** corresponds to hydroxytyrosol (HT); compounds **2** and **3** are intermediate derivatives in the synthesis described in the main text; compound **4** corresponds to HT-C6.

The synthesis of the hydroxytyrosyl alkyl ether **4** was carried out according to a slightly modified procedure described by Madrona et al. [8] (Figure 1).

Compound **2**: To a solution of hydroxytyrosol (1) (1.0 g; 6.48 mmol, 1.0 eq) in dry acetone (15 mL) were added K_2_CO_3_ (3.59 g; 25.9 mmol, 4.0 eq) and BnBr (1.56 mL; 12.96 mmol, 2.0 eq) and the mixture was refluxed for 24 h. After this time, the mixture was filtered over celite and concentrated over reduced pressure. The crude mixture was purified by flash column chromatography (silica gel, 20% of ethyl acetate in hexanes) to obtain 2-(3,4-bis(benzyloxy)phenyl)ethan-1-ol (2) (1.63 g, 75%) as a white solid.

Compound **3**: To a solution of **2** (0.355 g; 1.06 mmol, 1.0 eq) in DMSO (13 mL) were added KOH (0.420 g; 6.36 mmol, 6.0 eq) and 1-hexyliodide (0.48 mL; 3.18 mmol, 3.0 eq). The mixture was stirred until depletion of starting material, as judged by thin-layer chromatography. When the reaction was completed, it was neutralized with a 3.0 M aqueous HCl solution (15 mL) and then extracted with Et_2_O (3 × 20 mL). The organic layer was successively washed with saturated aqueous NaHCO_3_ solution (30 mL), water (30 mL) and brine (30 mL), dried over MgSO_4_ anhydrous, filtered and concentrated under reduced pressure. The crude product was purified by flash column chromatography (silica gel, hexanes 5% of ethyl acetate in hexanes 10% of ethyl acetate in hexanes) to afford **3** (0.254 g, 57%) as a colorless oil.

Compound **4**: To a degassed solution of compound **3** (0.254 g, 0.607 mmol) in 35% of ethanol in ethyl acetate (15 mL) was added Pd(OH)_2_/C (20 mg). This mixture was sequentially put under vacuum and H_2_ 10 times, finishing the cycle with H_2_. The reaction was then stirred under an H_2_ atmosphere at room temperature overnight to afford **4** (0.143 g, 99%) as a colorless oil, not requiring further purification.

### 2.2. Cell Culture and Treatments

Human umbilical vein endothelial cells (HUVECs) were used as in vitro EC model. This primary cell line was purchased from Lonza (Basel, Switzerland), and manufacturer recommendations were followed for maintenance media (EGM-2, purchased from Lonza, Basel, Switzerland) and maximum passage (pass 9). Human monocyte cell line THP-1 was obtained from American Type Culture Collection (ATCC) and maintained in RPMI 1640 media (Corning, MA, USA) supplemented with 10% fetal bovine serum (FBS; Capricorn Scientific, Ebsdorfergrund, Germany), a mix of 10,000 IU/mL penicillin and 10,000 μg/mL streptomycin (Corning, MA, USA), 2 mM L-glutamine (Merck, Darmstadt, Germany) and 0.05 mM β-mercaptoetanol (Merck, Darmstadt, Germany). Cell lines were grown in a 5% CO_2_ humid atmosphere at 37 °C. HT-C6 was dissolved in dimethyl sulfoxide (DMSO) at 100 mM stock solution and used in the different assays at the indicated doses in EGM-2 media; DMSO (vehicle) was used in an equivalent concentration in control conditions of each assay.

### 2.3. Cell Growth/Survival Assay

HUVECs (2 × 10^3^ cells) were incubated in MW96 plates under serial dilutions of HT-C6 (in quadruplicate). After 3 days of incubation, 10 µL of methylthiazol tetrazolium (MTT; Merck, Darmstadt, Germany) at a concentration of 5 mg/mL in PBS was added to wells, and then the plate was incubated for a further 4 h. 0.04 N HCl-2-propanol was used to dissolve the resulting formazan, and developed color was measured at 550 nm in an Eon microplate spectrophotometer (BioTek, Charlotte, VT, USA). The concentration of HT-C6 yielding 50% of cell growth in HUVECs was defined as its IC_50_ value at 3 days of treatment. Three independent replicates were performed for this assay.

### 2.4. Cell Cycle Analysis

HUVECs were grown to subconfluency in MW6 plates and then treated with HT-C6 at indicated doses. Cells treated with vehicle (DMSO) and 10 μM 2-methoxyestradiol (2-ME) were included as negative and positive control of apoptosis, respectively. Harvested cells were washed with 1% FBS, 10 mM HEPES in PBS and fixed in 70% ice-cold ethanol for 1 h at 4 °C. After washing; cells were incubated in propidium iodide (PI) staining solution (40 μg/mL propidium iodide, 0.1 mg/mL RNase-A and 2 mM EDTA in PBS supplemented with 1% FBS and 10 mM HEPES) during 30 min at 37 °C. FACS VERSE^TM^ flow cytometer and BD FACSuite software version 2.1.2 (BD Biosciences, Franklin Lakes, NJ, USA) were used for cell cycle analysis. Population of cells in G_0_/G_1_, S and G_2_/M phases of the cycle and the population in sub-G_1_ (fragmented DNA) were determined in each condition. Three independent replicates were performed for this assay.

### 2.5. Real-Time Quantitative PCR

Pretreatment of HUVECs with HT-C6 at indicated doses was performed 30 min before TNF-α induction (20 ng/mL). HT-C6 treatments were maintained for 6 h in presence of TNF-α, and then cells were harvested and RNA was extracted using Tri Reagent (Sigma-Aldrich/Merck, Darmstadt, Germany) and a Direct-zol RNA MiniPrep kit (Zymo Research, Irvine, CA, USA). RNA in samples were quantified in a NanoDrop One spectrophotometer (Thermo Fisher Scientific, Waltham, MA, USA), and a maximum of 500 nmol of RNA were retrotranscripted to cDNA using PrimeScript™ RT Master Mix (Takara Bio Inc., Kusatsu, Japan). TB Green Premix Ex Taq II (Takara Bio Inc.) and predesigned custom primers (KiCqStart, Sigma-Aldrich/Merck, Darmstadt, Germany/) were used to perform real-time quantitative PCR (RT-qPCR) assays in an Eco Illumina device (Illumina; San Diego, CA, USA). In each case, mRNA expression was relativized to *ACTB* gene (β-actin) expression, and values were normalized with respect to expression levels in the positive control condition (cells induced with TNF-α in the presence of DMSO) in order to calculate fold change. Three independent replicates of these assays were performed, with technical duplicates in each assay.

### 2.6. Immunofluorescence

Gelatin-coated coverslips placed in MW6 plates were used to grow HUVECs until subconfluence. Cells were pretreated with HT-C6 for 30 min and then induced with 20 ng/mL TNF-α for 15 min in presence of the compound. After fixation in formalin solution (Sigma-Aldrich/Merck, Darmstadt, Germany), cells were permeabilized in Triton X-100 0.5%-PBS and blocked in 5% BSA-PBS. RelA/p65 mouse mAb (sc-8008; Santa Cruz Biotechnology, Dallas, TX, USA) was used for incubation during 1 h at room temperature in a wet chamber. Coverslips were washed in PBS prior to incubation with Alexa Fluor 568-linked anti-mouse IgG antibody (A11004, Invitrogen, Thermo Fisher Scientific, Waltham, MA, USA). Then, 3 washes in PBS were performed and 1 mg/mL Hoechst 33342 was used for 5 min incubation. Coverslips were mounted in glass slides using 1:10 (vol-vol) glycerol-water solution. Fluorescence-confocal microscope Leica TCS SP8 MP and Leica Application Suite X software version 5.1.0 (Leica Microsystems, Wetzlar, Germany) were used for sample visualization and imaging capture. Fiji software (https://imagej.net/contribute/citing, accessed on 29 June 2023) [21] was used for quantification of nuclear/cytosolic signals in at least 5 images of each experimental condition. Three independent replicates were performed for this assay.

### 2.7. Western-Blot

HUVECs were pre-treated with HT-C6 at indicated doses for 30 min prior to TNF-α induction. For VCAM-1 detection, cells were incubated with TNF-α and HT-C6 for additional 8 h; in the case of total IKK/phospho-IKK and total p65/phospho-p65 detection, TNF-α induction in presence of HT-C6 treatment was performed for 15 min after pre-treatment. Cell lysates were performed in 0.5 M Tris-HCl, pH 6.8, 12% SDS, 10% glycerol and protein concentration was determined in samples using DC Protein Assay (Bio-Rad Laboratories; Hercules, CA, USA). Prior denaturalization at 95 °C, β-mercaptoethanol (5% final concentration) and bromophenol blue (0.2% final concentration) were added to samples. Moreover, 30 mg of total protein was separated by SDS-PAGE electrophoresis and transferred into nitrocellulose membranes. Blocking of the membranes was performed in 10% non-fatty dry milk in TBS-T (20 mM Tris pH 7.6, 137 mM NaCl, 0.1% Tween-20). Incubation with primary antibodies was carried out overnight at 4 °C: phospho-IKKα/β (Ser176/180; 16A6) rabbit mAb (#2697), IKKβ (D30C6) rabbit mAb (#8943), phospho-RelA/p65 (Ser536; 93H1) rabbit mAb (#3033) and α-Tubulin (DM1A) mouse mAb (#3873) antibodies were provided by Cell Signaling Technology (Danvers, MA, USA). VCAM-1 mouse mAb (sc-13160) and RelA/p65 mouse mAb (sc-8008) were purchased from Santa Cruz Biotechnology (Dallas, TX, USA). After washing in TBS-T, membranes were incubated for 1 h with secondary horseradish peroxidase-conjugated secondary antibodies at room temperature (donkey anti-rabbit IgG HRP-linked was from Merck (Darmstadt, Germany), and horse anti-mouse IgG HRP-linked was from Cell Signaling Technology). SuperSignal West Pico Chemiluminescent Substrate (Pierce; Rockford, IL, USA) was used for detection of immunoreactive bands in a Chemidoc XRS System (Bio-Rad Laboratories; Hercules, CA, USA). Fiji software (https://imagej.net/contribute/citing) [21] was used for densitometric quantification of the bands. Values were normalized as a ratio of phosphorylated/non-phosphorylated protein or total protein/α-tubulin, and positive control of induction (DMSO plus TNF-α) was used to relativize treatment conditions in each experiment. At least three independent replicates were performed of these assays.

### 2.8. Chemotaxis Assay

HUVECs were grown to subconfluency in MW6 plates and were then pre-treated for 30 min with different doses of HT-C6 in EBM-2 media (Lonza, Basel, Switzerland) supplemented with 0.2% bovine serum albumin (BSA). After that, induction with 20 ng/mL TNF-α was conducted. Conditioned media were collected after 16 h (overnight incubation), and centrifuged at 1500 rpm for 5 min to remove cellular debris. Concurrently, THP-1 cells were stained with 5 μg/mL calcein (Calbiochem, Merck, Darmstadt, Germany) for 30 min, and were then washed with PBS, centrifuged at 1500 rpm for 5 min and resuspended in EBM-2 medium. Next, 700 μL of conditioned media were placed on wells, onto which 8 μM FluoroBlok™️ Inserts (Corning, MA, USA) were placed. Finally, 200 μL of a THP-1 cell suspension (5 × 10^4^ cells) were seeded onto the inserts. The plaque was then incubated for 2 h, and fluorescence was measured in an FL600 fluorescence plate reader (BioTek, Charlotte, VT, USA) every 30 min. The signal was measured in arbitrary units (RFUs). In addition to negative and positive controls of TNF-α induction, negative controls of staining and chemoattraction were included in this experiment.

### 2.9. Adhesion Assay

10^5^ HUVECs per well were seeded in an MW24 plate the afternoon before the experiment, and cells were incubated overnight. HUVECs were then pre-treated with different doses of HT-C6 in EGM-2 media 30 min before induction with TNF-α, and cells were incubated for additional 6 h. Moreover, 1 h before the end of the treatments, THP-1 cells were stained with 5 μg/mL calcein (Calbiochem, Merck, Darmstadt, Germany) for 30 min, and were then washed with PBS, centrifuged at 1500 rpm for 5 min, and resuspended in EGM-2 medium. After a wash, HUVECs were co-cultured with the stained THP-1 for 1 h to allow monocyte adhesion. Subsequently, the co-culture was gently washed once with EBM-2 media to remove the non-adhered THP-1, and fresh EGM-2 media was added. Then, fluorescence was measured in an FL600 fluorescence plate reader (BioTek, Charlotte, VT, USA), and bright field and fluorescence images were taken in a fluorescence Nikon Eclipse Ti microscope (Nikon, Minato, Tokio, Japan).

### 2.10. Single Cell Monitoring of ROS Production

HUVECs were seeded in an MW96 plate at a density of 10,000 cells/well in a final volume of 200 μL. After overnight incubation, a pre-treatment with 0.5 mM hydrogen peroxide (H_2_O_2_) was conducted for 15 min to induce oxidative stress. After H_2_O_2_ removal, dyes were added at working concentrations indicated in Table 1 in presence or absence of different doses of HT-C6. Moreover, 1 h later, the time-lapse assay was conducted in the Operetta High Content Imaging System (PerkinElmer, Inc., Waltham, MA, USA) in which photographs were taken every 20 min for 5 h, maintaining normal incubation conditions of temperature and CO_2_.

Image analysis during assay was performed with Harmony software version 4.8 (PerkinElmer, Inc., Waltham, MA, USA). Subsequently, segmentation of the different cellular compartments marked by the dyes was performed, fluorescent signal was measured (fluorescence intensity, *FI*), the background signal of the *CellROX* dye (Thermo Fisher Scientific, Waltham, MA, USA) was subtracted, and the following processing step was performed:

$$\bar{X}FI=\bar{X}{CellROX}_{ROS\;Signaling}-\bar{X}{CellROX}_{Whole\;Cell}$$

Finally, the images obtained from the Operetta were analyzed and processed using FIJI software (https://imagej.net/contribute/citing) [21].

### 2.11. Statistical Analysis

At least three independent experiments were performed in all assays, and GraphPad Prism software version 9.0.0 was used to perform statistical analyses of obtained results. One-way ANOVA and Dunnett’s multiple comparisons test, and two-way ANOVA followed by Turkey’s multiple comparisons test were performed as indicated in figure captions; the Shapiro–Wilk normality test was conducted to assess normal distribution; *p* values < 0.05 were considered statistically significant. *p* values were represented as * *p* < 0.05, ** *p* < 0.01, *** *p* < 0.001, **** *p* < 0.0001.

## 3. Results

### 3.1. Short-Time Treatment of HT-C6 Does Not Affect EC Cell Cycle Progression

**Figure 2:**
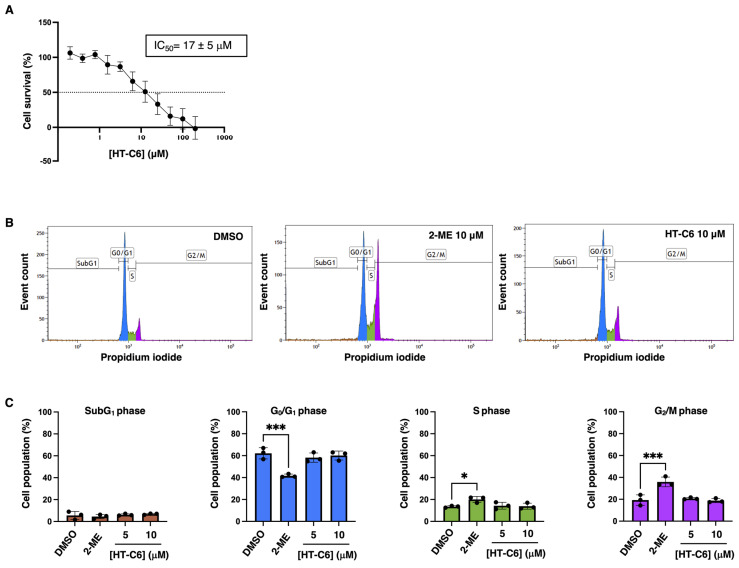
Effect of HT-C6 on survival and cell cycle of HUVECs. (**A**) Survival curves of HUVECs after 72 h treatment with HT-C6 measured by MTT assay; IC_50_ is indicated for these conditions (**B**) Cell cycle profiles of HUVECs measured by propidium iodide (PI) staining; conditions with DMSO (negative control), 10 μM 2-methoxyestradiol (2-ME; positive control of cell cycle impairment) or 10 μM HT-C6 are showed. (**C**) Sub-populations of cells (expressed in percentage of total population) in the subG_1_ (orange), G_0_/G_1_ (blue), S (green) and G_2_/M (purple) phases of the cell cycle after treatment with DMSO, 2-ME or different doses of HT-C6. Means and standard deviations of at least three independent experiments are represented. Statistical analysis was performed by one-way ANOVA + Dunnett’s multiple comparisons test; * *p* < 0.05, *** *p* < 0.001.

The viability of human endothelial cells in the presence of HT-C6 was assessed after 72 h of treatment under increasing doses of the compound. The obtained curve indicated that HT-C6 reduced cell survival with an IC_50_ value in the low micromolar range (17 μM) (Figure 2A). Interestingly, this value was more than two-fold higher than the one reported by us for bovine aortic endothelial cells (BAEC, 7.5 μM) [10]. Since HT-C6 was reported to induce apoptosis in BAEC at doses close to its IC_50_ [10], we analyzed the cell cycle profile of HUVECs treated with this compound at a concentration lower than the calculated IC_50_, and in a short time schedule, trying to establish a sub-lethal experimental condition for HT-C6 in these cells. As shown in Figure 2B, 10 μM of HT-C6 did not affect cell cycle progression in HUVEC after 6 h, as it did the positive control (2-ME 10 μM), which induced the accumulation of cells in S/G_2_/M phases. Quantitative data indicated that any of the phases of the cell cycle were significantly affected by HT-C6, and fragmented DNA was not detected at the assayed conditions (Figure 2C).

### 3.2. HT-C6 Impairs the Expression of Adhesion Molecules in ECs Induced by TNF-α

**Figure 3:**
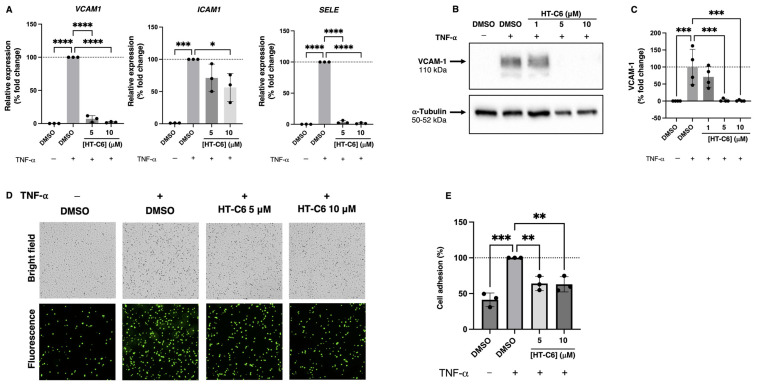
Effect of HT-C6 on adhesion molecules expression in TNF-α-induced HUVECs. (**A**) qPCR-measured relative expression of *VCAM1*, *ICAM1* and *SELE* genes in HUVECs under indicated treatments for 6 h; in each case, expression was relativized to the β-actin gene (*ACTB*) and normalized to the TNF-α-induced DMSO control. (**B**) Representative Western-blot images of vascular adhesion molecule 1 (VCAM-1) and ⍺-tubulin levels in HUVECs under indicated treatments for 8 h; (**C**) Densitometric quantification of VCAM-1 and α-tubulin bands in (**B**) and two additional independent experiments; VCAM-1 signals were relativized to α-tubulin and normalized to the TNF-α-induced DMSO control. (**D**) Representative images (upper, bright field; lower, fluorescence; 4× magnification) of adhesion assay performed with calcein-labelled THP-1 onto HUVECs monolayer under different experimental conditions; (**E**) Quantification of adhesive cells relative to TNF-α-induced DMSO control condition (100%). Means and standard deviations of at least three independent experiments are represented in (**A**,**C**,**E**). Statistical analysis was performed by one-way ANOVA + Dunnett’s multiple comparisons test; * *p* < 0.05, ** *p* < 0.01; *** *p* < 0.001; **** *p* < 0.0001.

The ability of HUVEC to activate a transcriptional response induced by the pro-inflammatory molecule TNF-α was studied in different experimental conditions. Thus, HT-C6 was included shortly before the TNF-α induction of ECs (30 min pre-treatment followed by 6 h of treatment plus induction), and then the expression of key genes involved in EC inflammatory response was measured. In the absence of HT-C6, TNF-α induced the expression of *VCAM1*, *ICAM1* and *SELE* in ECs, genes encoding vascular cell adhesion molecule 1 (VCAM-1), intercellular adhesion molecule 1 (ICAM-1) and E-selectin. Our data clearly show that the treatment of the cells with HT-C6 abrogated the TNF-α-induced response, with a strong inhibition in *VCAM1* and *SELE* expression (Figure 3A), indicating an anti-inflammatory effect in ECs by prevention of the transcriptional program associated with the pro-inflammatory context in the endothelium. Additionally, the presence of VCAM-1 was analyzed by Western-blot after TNF-α induction, and, according to the observed inhibition of *VCAM* expression, HT-C6 strongly suppressed the presence of VCAM-1 in ECs (Figure 3B,C). The functional impairment of the inflammatory program was tested in an ECs–monocytes adhesion assay, in which HUVECs were induced with TNF-α in the presence or absence of different doses of HT-C6 prior to incubation with calcein-labeled THP-1 human monocytes. As shown in Figure 3D,E, HT-C6 reduced the adhesion of THP-1 to ECs, as a consequence of the decreased presence of adhesion molecules in these cells.

### 3.3. Chemoattractant Potential of ECs Is Reduced in Presence of HT-C6

**Figure 4:**
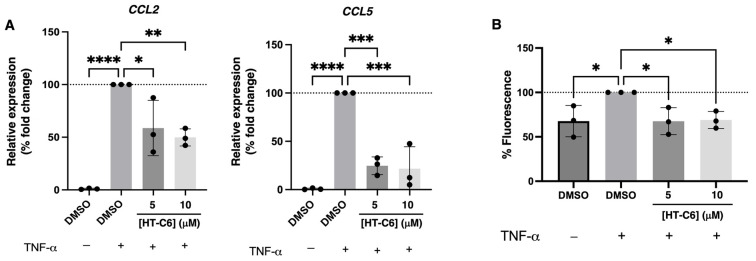
Effect of HT-C6 on chemoattractant molecules expression in TNF-α-induced HUVECs. (**A**) qPCR-measured relative expression of *CCL2* and *CCL5* genes in HUVECs under indicated treatments for 6 h; in each case, expression was relativized to the β-actin gene (*ACTB*) and normalized to the TNF-α-induced DMSO control. (**B**) Relative quantification of the fluorescence emitted by calcein-stained monocytes migrated through FluoroBlok™ Inserts towards conditioned media of pre-treated and TNF-α-induced HUVECs under the conditions indicated in the graph. Data are expressed in percentage of fluorescence and normalized to the TNF-α-induced DMSO control. Means and standard deviations of three independent experiments are represented. Statistical analysis was performed by one-way ANOVA + Dunnett’s multiple comparisons; * *p* < 0.05, ** *p* < 0.01, *** *p* < 0.001, **** *p* < 0.0001.

In addition to the activation of adhesion molecules-encoding genes, TNF-α induced the expression of *CCL2* and *CCL5* in ECs, genes encoding C-C motif chemokine ligand 2 (CCL2, also known as monocyte chemoattractant protein 1, MCP1) and C-C motif chemokine ligand 5 (CCL5, also known as RANTES). The treatment of HUVEC with HT-C6 prevented the TNF-α-mediated activation of *CCL2* and *CCL5* expression in ECs (Figure 4A). Since CCL2 and CCL5 secreted by endothelium exert chemoattractant properties for immune cells such as monocytes, T cells and eosinophils, the decreased expression of these genes in the presence of HT-C6 was expected to result in a reduced chemotactic potential of HT-C6-treated ECs. As it is shown in Figure 4B, the migration of human monocytes THP-1 was stimulated by a conditioned medium of TNF-α-induced HUVECs, but this migration was decreased when HUVECs were treated with HT-C6 (Figure 4B), indicating the impaired chemoattractant ability of ECs in presence of the compound.

### 3.4. NF-κB Pathway Is Inhibited by HT-C6 in ECs

**Figure 5:**
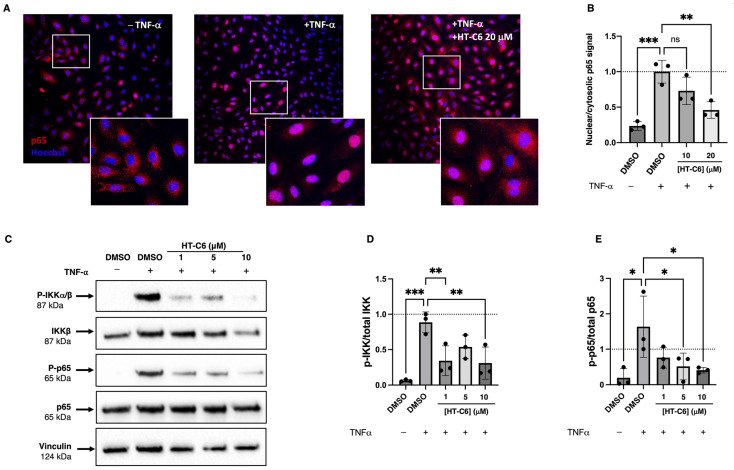
Effect of HT-C6 on NF-κB pathway in HUVECs. (**A**) Representative confocal images of p65 (red) merged with Hoechst (blue) in HUVECs under indicated treatments (20× magnification and digital zoom in insets); (**B**) The relative nuclear/cytosolic p65 signal was measured in confocal images of three independent experiments and means and standard deviations are represented. Statistical analyses were performed through one-way ANOVA + Dunnett’s multiple comparisons test; ** *p* < 0.01, *** *p* < 0.001. (**C**) Representative Western-blot images of IKK⍺/β, p65 and their phosphorylated forms (P-IKK⍺/β, P-p65 respectively). Vinculin (cytosolic protein) was included as loading control. (**D**,**E**) Densitometric quantification of (**C**) and three additional independent experiments, bands of phosphorylated proteins were relativized to the band of their corresponding total protein and normalized to the negative control unstimulated cells. Means and standard deviations are represented, and statistical analyses were performed through one-way ANOVA + Dunnett’s multiple comparisons test; * *p* < 0.05, ** *p* < 0.01, *** *p* < 0.001.

NF-κB pathway is a master regulator of the inflammatory response in ECs, activating the expression of key pro-inflammatory genes. Thus, the effect of HT-C6 in the NF-κB pathway was explored in TNF-α-induced HUVECs. As shown in Figure 5A, TNF-α elicited a fast nuclear translocation of the NF-κB subunit p65, which was abrogated in the presence of HT-C6 (Figure 5A,B). Trying to identify the impairment in the NF-κB pathway resulting in decreased p65 import to the nucleus, the phosphorylation of IKK complex was investigated since it directly regulates the phosphorylation (and hence degradation) of the NF-κB inhibitory protein IkBα, responsible for its retention in the cytosol. According to the observed impaired nuclear translocation of p65, the treatment of ECs with HT-C6 decreased the phosphorylation of the IKK complex in response to TNF-α (Figure 5C,D). In addition, the phosphorylation of p65 (an essential step for the activation of its binding to DNA) was measured, detecting a reduction in phosphorylated-p65 in ECs treated with HT-C6 (Figure 5C,E). Altogether these results pointed to the inhibitory effect of HT-C6 in the NF-κB pathway under TNF-α induction in ECs.

### 3.5. HT-C6 Prevents ROS Production in ECs

**Figure 6:**
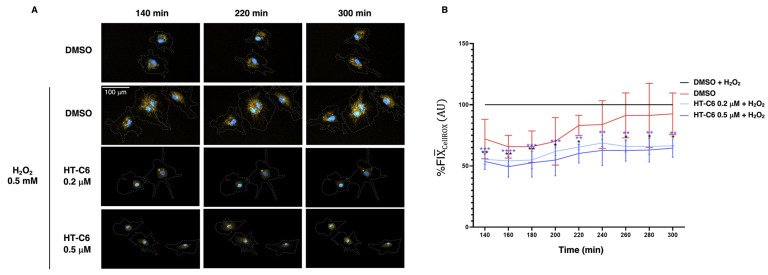
Effect of HT-C6 in ROS production in HUVECs. (**A**) Operetta images (20× magnification, scale bar 100 μm) with the combination of dyes used for nuclear staining (Hoescht, blue), ROS signaling (CellROX, orange) and plasma membrane visualization (CellMask, dashed line). Segmentation was performed using Harmony software (version 4.8). The control H_2_O_2_ + DMSO and control DMSO conditions consisted of 116 cells and 126 cells, respectively, while the HT-C6 treatment groups at concentrations of 0.2 µM and 0.5 µM had 84 and 68 cells, respectively; (**B**) Relative percentage quantification, compared to the control (H_2_O_2_ DMSO), of the fluorescence intensity (FI) emitted in the HT-C6-treated groups (0.2 and 0.5 µM). A two-way ANOVA test was performed, followed by Turkey’s multiple comparisons test, * *p* > 0.05, ** *p* > 0.01, *** *p* > 0.001, **** *p* > 0.0001.

In addition to the response of ECs to inflammatory stimuli, the impairing effects of HT-C6 in NF-κB pathway activation could be modulating other cellular processes since this pathway activates the expression of multiple genes, not only associated with inflammation. This is the case of genes involved in redox control, and the role of NF-κB in oxidative stress has been largely studied, pointing to a dual pro- and anti-oxidant activity depending on the cell type and context [22]. The antioxidant effect of HT-C6 was previously described in several non-cellular models [18], suggesting a protective activity against oxidative stress in cells. In order to validate the antioxidant activity of HT-C6 in ECs, the intracellular ROS production was monitored in HUVECs by a single-cell real-time approach. Since the cell density used in this assay was low (necessary for single-cell monitorization), the concentration of HT-C6 in the treatments was reduced compared to other assays in order to avoid undesired effects due to a higher sensibility of individual ECs. As shown in Figure 6, H_2_O_2_-challenged HUVECs increased the signal for CellROX dye, indicating a higher presence of ROS under this condition. However, the treatment with low doses of HT-C6 significantly reduced the presence of ROS after the H_2_O_2_ challenge in ECs, denoting an antioxidant effect upon oxidative stress.

## 4. Discussion

The study of natural compounds as drug candidates is an expanding field of research in biomedicine and pharmacology since the plethora of secondary metabolites of plants, fungi or microorganism represents a valuable source of new molecules with potential therapeutic use [23]. In this context, the identification of bioactive molecules naturally present in foods has been suggested as part of the underlying mechanism of health-promoting properties of certain dietary patterns, such as the Mediterranean Diet (MD) [1,24]. EVOO emerges as the main exponent of the MD, and multiple components of EVOO, including phenolic compounds, have been described as bioactive molecules with interesting effects in cellular processes [2,25]. However, natural compounds could exhibit certain limitations attending to their pharmacokinetic properties, representing a handicap in the development of new drugs, which can be surpassed by the synthesis of derivatives with improved activity or pharmacokinetics [6].

Previous studies from our group explored the optimization of the antiangiogenic properties of the phenolic compound hydroxytyrosol, naturally present in EVOO, studying a series of alkyl ether derivatives with more lipophilic behavior, from which HT-C6 emerges as the most active HT derivatives studied [10]. In addition to its antiangiogenic activity, the potential role of HT-C6 in vascular damage prevention was previously reported, showing its anti-platelet effect in vitro and in vivo [11,12] and suggesting the potential therapeutic interest of this compound in atherosclerosis, although its role in ECs has not been investigated in this pathological context before. Data presented in this work show for the first time the anti-inflammatory activity of HT-C6 in the endothelium, preventing the inflammatory response elicited by TNF-α in ECs and impairing monocyte recruitment and adhesion by a mechanism involving NF-κB pathway inhibition.

One of the first phenotypic changes detected in activated endothelium during atherosclerosis is the expression of genes encoding inflammatory-related proteins in response to pro-inflammatory stimulus, which is involved in the progression of the atheroma favoring the infiltration of immune cells into the intima layer [26,27]. The observed reduction in TNF-α-induced expression of *VCAM1*, *ICAM1* and *SELE* in ECs in the presence of HT-C6 correlated with a poor ability of these cells to adhere monocytes in vitro, probably due to the decreased exposition of adhesion molecules in the cell surface, corroborated by our data showing downregulation of VCAM-1 in HUVECs treated with HT-C6. In addition, the compound decreased the migration of monocytes towards conditioned media of TNF-α stimulated ECs, as a consequence of the detected inhibition of *CCL2* and *CCL5* expression. Together these data suggest that HT-C6 could interfere with the chemoattractant and adhesive abilities exhibited by activated endothelium during atheroma formation, impairing the rolling of circulant immune cells and hence their infiltration into the subendothelial intima layer.

Although the exact mechanism of action of HT-C6 in reducing the endothelial inflammatory response is not fully understood, our results clearly show that this compound is able to inhibit the activation of the NF-κB pathway in ECs, a master signaling cascade leading to an inflammatory response. The switching on of this pathway drives a transcriptional response that activates the expression of a high number of genes involved in inflammation, such as *VCAM1*, *ICAM1*, *SELE*, *CCL2* and *CCL5*, which are targets of NF-κB [14,16]. Our results show that treatment of ECs with HT-C6 downregulates the phosphorylation of the IKK complex after TNFα induction, one critical point in the intracellular activation of the NF-κB pathway. During canonical activation, the phosphorylated IKK complex drives the proteasomal degradation of IkBα, the cytosolic inhibitor of NF-κB, leading to the release of this transcription factor and its consequent translocation to the nucleus, where NF-κB activates its target genes. Indeed, in this work, we show that HT-C6 partially reduces nuclear localization of the NF-κB subunit p65, and this effect is observed after short treatment of ECs with the compound, suggesting a direct activity of HT-C6 in the modulation of the pathway. Other HT derivatives, but not HT-C6, have been described to target key signaling pathways in prostate cancer cells, such as MAPK, AKT, JAK/STAT, TFGβ and NF-κB pathways, although this effect was assessed in longer treatment conditions (24 h), and the impairment of NF-κB nuclear translocation was not evaluated [28]. Together with the observed impairment in NF-κB nuclear import, our data indicate that HT-C6 decreases the phosphorylation of p65 in ECs. This observation suggests that the transactivation potential of NF-κB is reduced upon treatment with the compound since the presence of phosphorylation sites in p65 has been correlated with the regulation of its ability to bind promoters of target genes [29]. The strong inhibition of NF-κB target genes observed in HUVECs induced with TNF-α in the presence of HT-C6 could be explained considering the double effect described in our work for this compound: (i) reduction in nuclear localization of NF-κB, and (ii) inhibition of p65 phosphorylation, two molecular events that would determine the observed abrogation of ECs inflammatory response by HT-C6.

The pleiotropic bioactivity of HT-C6 reported in this work and in the previous literature represents an interesting potential use of this HT-derived compound in the prevention of vascular damage at the onset of atherosclerosis and during different stages of pathogenesis. Indeed, targeting the endothelial inflammatory response by impairment of NF-κB pathway activation, HT-C6 would prevent the infiltration of immune cells into the vascular wall, one of the main events in the formation of atheromas [27]. The antioxidant activity of HT-C6 in ECs was manifested in our work, although the technique used for ROS monitorization limited the concentration of HT-C6 since individual ECs (losing cell–cell contact typical of a monolayer) are more sensitive to drug treatments. These data correlate with the proposed antioxidant role of this compound in non-cellular systems [18]. In contrast, preliminary results of our laboratory detected that HT-C6 could induce ROS production in ECs in the absence of H_2_O_2_ challenge (data non-shown), which suggests that HT-C6 could act as an antioxidant or pro-oxidant molecule depending on the redox status of the cell. This observed duality is in accordance with the reported pro-oxidant role of hydroxytyrosol in tumoral contexts [30], and it deserves to be explored in future works. The production and release of ROS in atheroma is an essential event in the progression of the disease, and the ability of HT-C6 to reduce ROS levels in ECs under a pro-oxidant context would contribute to the amelioration of the pathogenesis. Together with these activities, we previously described the antiangiogenic potential of HT-C6 [10]. The formation of new vessels from *vasa vasorum* in the arterial wall is observed in the late stages of the atheroma progression as a consequence of the activation of angiogenesis by proangiogenic factors released by immune cells [31,32]. The use of antiangiogenic drugs in this context has been proposed as a therapeutic strategy in atherosclerosis [33,34,35]. In this scenario, the antiangiogenic, anti-inflammatory and antioxidant effects of HT-C6 in endothelium represent a promising therapeutic option in atherosclerosis that deserves to be explored in future studies in vivo.

## 5. Conclusions

Targeting inflammatory response in the endothelium is a therapeutic option in the control of atherosclerosis, mainly in its early stages, and the use of potential drugs that could impair this response is an interesting approach in pharmacologic research. Our results support the pharmacological interest of HT-C6, an alkyl ether derivative synthesized from the natural phenolic compound hydroxytyrosol, as an inhibitor of the endothelial inflammatory response, opening the way to its further development as an anti-inflammatory molecule in the context of atherosclerosis. Despite the promising anti-inflammatory bioactivity defined in this work for HT-C6, future studies are needed to explore the putative therapeutic role of this compound in in vivo models of early atherosclerosis.

## Figures and Tables

**Table 1:** Fluorescent dyes ^1^ used in ROS detection assay and methodology data.

Dye	Target	Working Concentration	Exposure Time and Height Excitation	Emission Range (exc/ems, nm)	Channel	Reference
Hoechst	DNA	2.5 μm/mL	10 ms–1 μm	360–400/410–530	UV Filter	H1399
CellROX © Deep Orange	ROS	50 μM	200 ms–3 μm	520–550/560–630	Red Channel (TRITC)	C10443
CellMask^TM^ Deep Red	Plasma membrane	0.25 μm/mL	50 ms–1 μm	620–640/650–760	Far-Red Channel (CYT5)	C10046

^1^ All dyes were purchased from Invitrogen^TM^ (Thermo Fisher Scientific, Waltham, MA, USA).

## Data Availability

Data are available within the article.
